# Expansion-Induced Crack Propagation in Rocks Monitored by Using Piezoelectric Transducers

**DOI:** 10.3390/s20216054

**Published:** 2020-10-24

**Authors:** Chi-Hyung Ahn, Dong-Ju Kim, Yong-Hoon Byun

**Affiliations:** 1 School of Electrical, Electronics and Communication Engineering, Korea University of Technology and Education, Cheonan-si 31253, Korea; chahn@koreatech.ac.kr; 2 School of Agricultural Civil & Bio-Industrial Engineering, Kyungpook National University, Daegu 41566, Korea; kyrix1028@knu.ac.kr

**Keywords:** crack propagation, expansion, heater, piezoelectric transducer, rock fracture

## Abstract

The objective of this study is to develop a new vibration-free excavation method based on vermiculite expansion for rock cracking and to evaluate the performance of the heating system via elastic wave monitoring. Natural vermiculites expand rapidly in volume when heated above 800 °C. MgO powder is used to evenly transmit the surface temperature of a heater rod, which can attain high temperatures rapidly, to the vermiculites. The insertion direction of the heater rod greatly affects the expansion pressure. Three cuboid rock specimens are prepared and equipped with the heating system at different hole-to-face distances. Crack propagation is monitored by a pair of disk-shaped piezoelectric transducers. For short hole-to-face distances, the wave velocity and maximum amplitude rapidly decrease after certain time. For the greatest hole-to-face distance, the shear wave velocity remains constant during the test, while the maximum amplitude decreases after a certain time. The time taken for the velocity and amplitude of the shear waves to decrease reasonably corresponded to that taken for detectable crack propagation to occur on the surface of the rock specimen. The proposed method and materials may be useful from the viewpoints of rapid expansion, economy, and crack control.

## 1. Introduction

Various excavation methods are employed for constructing tunnels and underground spaces; drilling and blasting is one of the common excavation methods. However, the propagation of the stress waves, which are induced during the blasting process, to the ground surface may cause vibration and noise. To minimize such vibration, controlled blasting methods such as line-drilling and pre-splitting are used at the tunnel perimeter [1]. Nevertheless, the blast-induced stress wave can still propagate through the connected area between empty drilling holes.

Various excavation methods were developed to reduce the blast-induced vibration. Jeng et al. [2] suggested a new technology using an abrasive water jet for cutting rocks. Experimental and numerical studies on the effects of precutting the tunnel perimeter by an abrasive water jet on blast-induced vibration showed that such a jet system can be useful as an auxiliary tool for creating a free surface along the tunnel perimeter [3,4]. As another vibration-reduced excavation method, namely, the wire-saw cutting technique was proposed by Gustafsson [5]. Lee et al. [6] investigated the effect of a pre-cut discontinuity on the attenuation of blast-induced vibration. The cutting performance of a wire-saw cutting machine was reported by Lee et al. [7]; however, only rectangular rock masses can be obtained by this method. For a more rounded tunnel section, more drilling holes have to be drilled, which is time-consuming. Although the use of tunnel-boring machines (TBMs) is considered a vibration-reduced excavation method, a small amount of vibration is created [8]; further, the use of TBMs for a limited section may be uneconomical.

Vibration-free excavation methods using non-explosive expansion materials have been widely used in rock quarry mining [9,10,11]. When non-explosive expansion materials are mixed with water, the lime in the material is hydrated, releasing heat. The non-explosive expansion materials in a slurry state are poured into pre-drilled holes in rocks. These materials then generate the expansive pressure, with their volume nearly doubling. Vibration-free excavation methods using such materials are safe and manageable. However, for lime-based expansion material, a few hours are required to generate the incremental static load to the drilled hole. Recently, a new vibration-free excavation method based on the expansion of vermiculite was developed for rock cracking [12]. Upon heating, the individual platy particles of the vermiculite can rapidly expand perpendicular to the cleavage planes up to 20–30 times their original volume [13,14], and the pressure induced by vermiculite expansion is sufficient for fracturing rocks [15]. However, as the cartridge heater developed by Ahn and Hu [12,15] was vulnerable to rock fracture, there is a need for a more durable heating system to induce rock fracture.

Macroscopic brittle failure of rock induced by stress and thermal conditions can be observed easily by the naked eye. However, before brittle failure, microcracking can be detected by several methods such as a reflection technique based on acoustic emission, volumetric strain measurements, and elastic wave velocity monitoring [16]. Elastic wave-based testing methods are widely used to investigate the elastic wave characteristics of various geomaterials [17,18,19,20,21,22]. It is well known that the elastic wave velocities in a solid will be reduced by the presence of cracks [23,24]. Byun et al. proposed a new method to predict the crack density in porous-cracked rocks using the elastic wave velocities [17]. However, few attempts have been made to better characterize crack propagation in large rock specimens based on the elastic wave characteristics under the mechanical expansion of brittle rock.

This paper describes the development of a modified heating system based on vermiculite expansion for rock fracture and the monitoring of the performance of the heating system by using elastic waves. First, the material properties of vermiculite and its expansion characteristics are introduced. This is followed by a description of the modified heating system developed in this study. The process of the vibration-free excavation method based on the expansion of vermiculite is then explained. The piezoelectric transducers and experimental setup for elastic wave measurement are described. Furthermore, the analysis of the elastic waves propagated through three different large rock specimens using piezoelectric transducers is described. The elastic wave characteristics and elapsed time to crack propagation are discussed according to the distance from the borehole to the free face.

## 2. Materials and Methods

### 2.1. Expansion Materials

**Figure 1:**
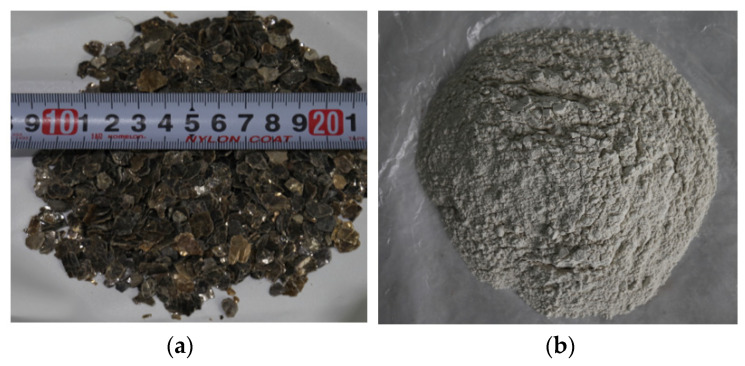
Photos of the expansion-induced materials: (**a**) vermiculite flake; (**b**) MgO powder.

Vermiculite, a multilayer silicate mineral, consists of hydrated silicates of aluminum, magnesium, iron, calcium and potassium. When the vermiculite is heated rapidly over 800 °C, its volume increases up to 20–30 times. Note that the expansion depends on vermiculite composition [25]. Expanded vermiculite is used for building materials, insulation, and environmental protection because it has lightweight, non-toxic, chemical inertness, and flame-retardant properties. Figure 1a shows a vermiculite flake. The natural mineral contains three types of water: absorbed water, interlayer water, and crystal water. When the vermiculite is rapidly heated, the moisture in its layers evaporates into water vapour, thereby generating a burst of pressure within the vermiculite. As the pressure is released, the vermiculite peels and then expands. The proposed vibration-free excavation method is based on the expansion pressure of the vermiculites to crack the rock without a chemical explosion. To increase the total expansion pressure, all the vermiculite flakes in a hole should be heated almost simultaneously. It is important that heat energy is evenly distributed among vermiculite flakes from the surface of a heater rod, which acts as a heat source, along the central axis of the hole. In this study, MgO is used to pass heat to all vermiculite flakes thoroughly. MgO, a compound of magnesium and oxygen, absorbs water and carbon dioxide from the air. MgO is chosen owing to its high insulation property (1 × 10^17^ Ω·cm) and high thermal conductivity (≥ 40 W/mK). The ratio of the amount of the vermiculite and MgO was experimentally determined [12,15].

### 2.2. Heating System

**Figure 2:**
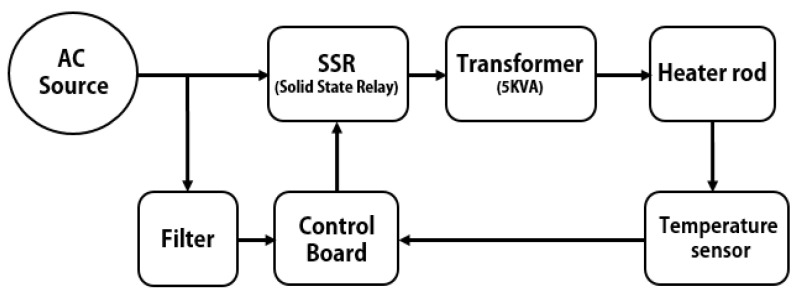
Block diagram of the heating system. The arrow lines indicate the direction of operation of devices.

In this study, a modified heating system was developed to overcome the limitation of the inability of increasing the temperature rapidly in early research [12]. Figure 2 shows the block diagram of the newly developed heating system. The solid state relay (SSR) delivers power from the alternating current (AC) source to the transformer while controlling the temperature of the heater rod. The transformer adjusts the voltage ratio of input and output power so that the system has a high output current, which can cause a rapid rise of temperature of the heater rod. The control board controls the SSR by measuring the value of the input temperature sensor and displays the current temperature and set temperature. The noise filter placed between the AC source and the control board removes the harmonic noise in the control board.

**Figure 3:**
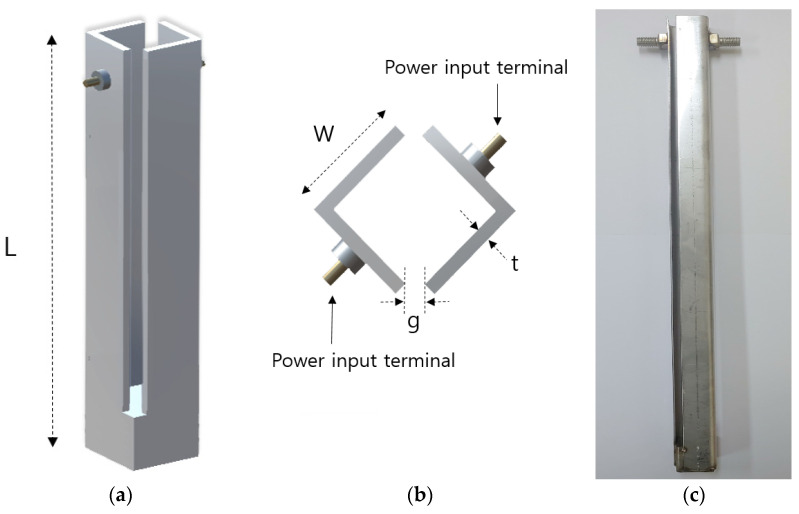
Structure of the heater rod: (**a**) side view (L = 500 mm); (**b**) top view (W = 2 mm, t = 0.1 mm, g = 0.3 mm); (**c**) photo of the heater.

**Figure 4:**
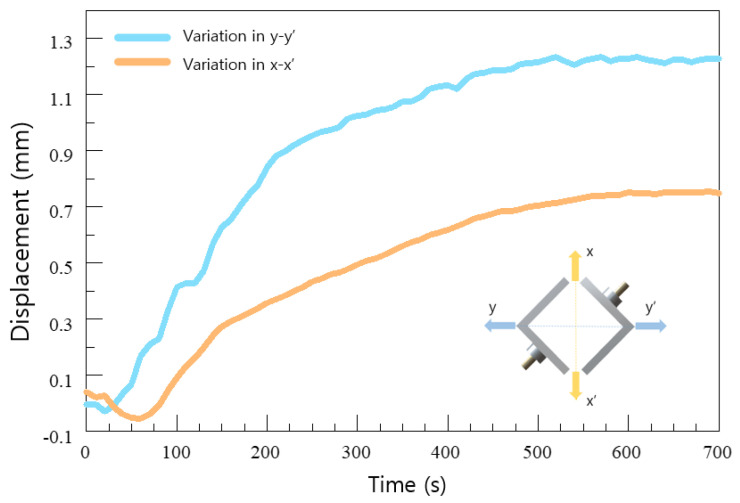
Displacement measured in a laboratory expansion test.

The structure of the heater rod was designed as shown in Figure 3 to withstand temperature rise in a short time. The rectangular structure of the heater controls the direction of the expansion pressure. To verify the direction of expansion pressure induced by the structure, a light-emitting diode (LED) sensor was used to measure the value of displacement according to the direction of a stainless steel tube, which was used instead of a drilled hole in natural rock in an early laboratory expansion test [12]. In Figure 4, the maximum displacement of the stainless steel tube was 1.22 mm in the y-y’ direction and 0.75 mm in the x’ direction.

### 2.3. Process of Expansion-Induced Fracturing

**Figure 5:**
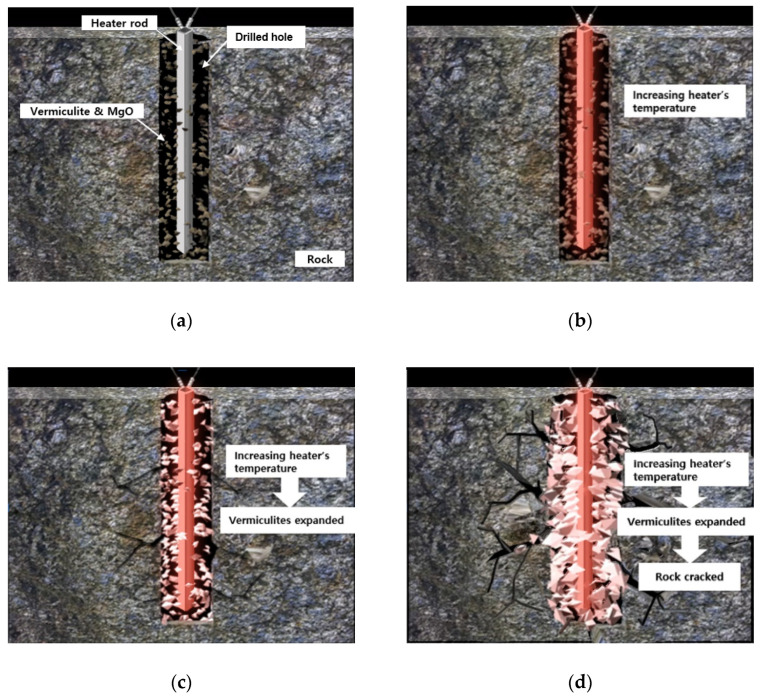
Conceptual drawings of process of the expansion-induced crack method: (**a**) setup of the heating device and materials in the drilled hole; (**b**) increasing the temperature of the heater rod; (**c**) vermiculite expansion; (**d**) cracking of the rock. The drawings are not to scale.

Figure 5 illustrates the process of the expansion-induced fracturing method. The first step is the setting up of a heater rod and expansion materials in a drilled hole of rock, as shown in Figure 5a. Once vermiculite flakes and MgO powers are evenly mixed and inserted into the hole along with the heater rod, the heating system is connected to the heater rod for operation. In the second and third steps, the temperature of the heater is raised (Figure 5b), and the vermiculite flakes are expanded (Figure 5c). The temperature of the heater is set to the maximum of 950 °C; this temperature is generally attained within 3 min after system operation. Figure 5d illustrates the final step in which the rock cracks because of the expansion pressure of the vermiculites. This whole process of the vibration-free excavation is completed within 30 min.

### 2.4. Rock Specimens

**Figure 6:**
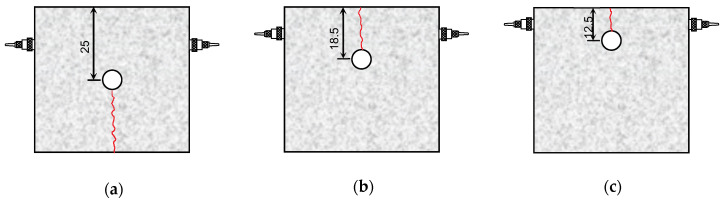
Schematic drawings of three rock specimens configured with transducers at hole-to-face distances of: (**a**) 25 cm; (**b**) 18.5 cm; (**c**) 12.5 cm. The numbers are in centimeter units.

Three cuboid specimens of dimensions 500 (length) × 500 (width) × 400 (height) mm were prepared for the application of the heat-based expansion technique. Holes of depth 350 mm and diameter 50 mm were drilled in each rock specimen. Figure 6 shows the holes drilled at three hole-to-face distances: 125, 185, and 250 mm. The rectangular heater was inserted into the drilled hole, and then, the space was filled with vermiculate and MgO. After installing this system, expansion tests were performed. In these tests, the temperature of the heater started increasing and then remained constant around 950 °C. Using the core samples for the tested rock, an unconfined compressive strength test and a Brazilian test were performed: the values of mean and standard deviation of unconfined compressive strength were 211 and 8.3 MPa, respectively, and the tensile strength was 13.6 MPa. The average shear wave velocity and the standard deviation estimated in the rock core samples were 2473 and 50 m/s, respectively.

### 2.5. Elastic Wave Measurement System

**Figure 7:**
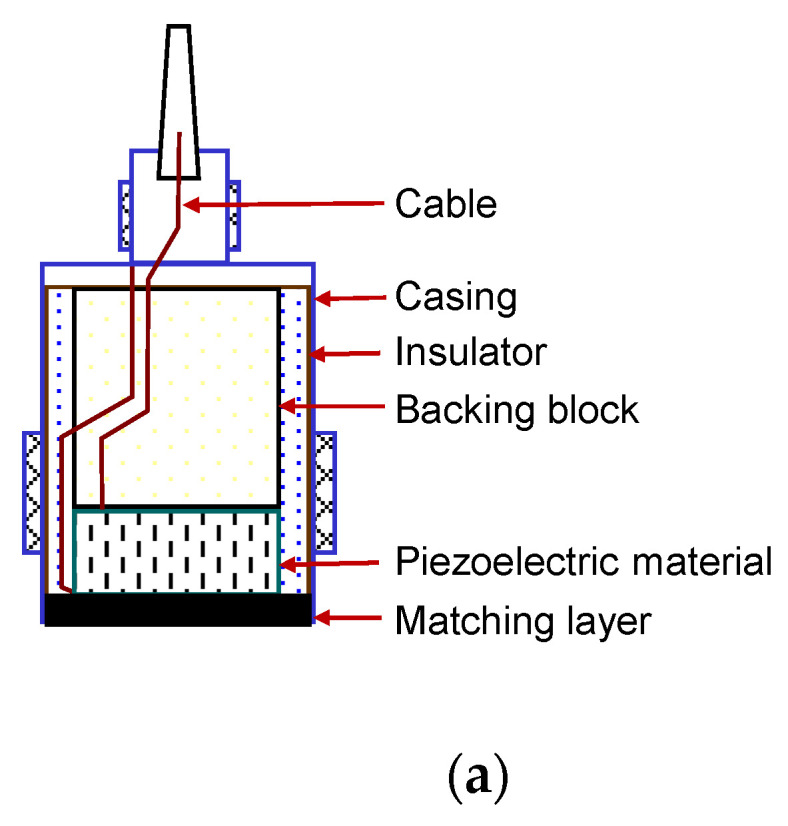

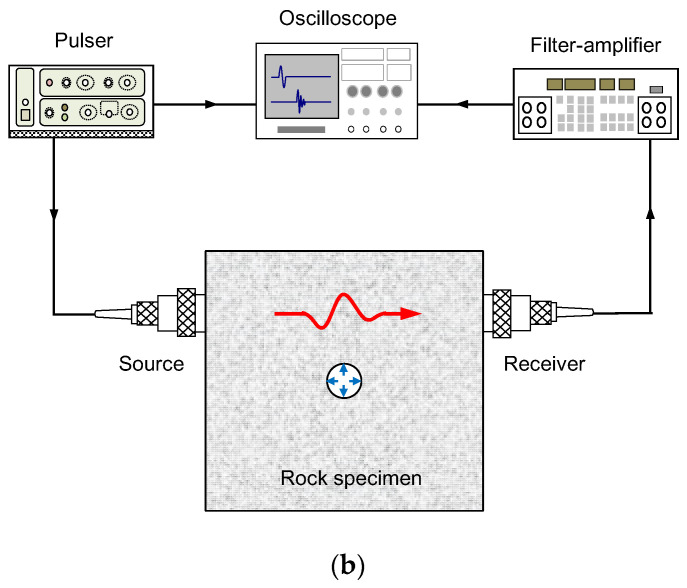
Schematic drawing of the wave measurement system: (**a**) piezoelectric transducer; (**b**) electronic devices with the rock specimen.

A pair of disk-shaped piezoelectric transducers was used to monitor the crack propagation in a rock specimen. A pulse-transmission method using the two piezoelectric transducers was employed to directly estimate the dynamic properties of a material placed between the two transducers. Figure 7a shows the structure of the piezoelectric transducers (Ultran, SWC75-0.05, Boalsburg, PA, USA), which consist of a piezoelectric element, backing block, matching layer, and casing. When a voltage is applied, the piezoelectric elements generate mechanical vibration. Conversely, the piezoelectric elements generate electric energy when they are deformed by external mechanical excitation. The backing block is effective for controlling the extent of ringing, and the impedance of the backing block should match that of the piezoelectric element to minimize internal reflections in the transducer. The matching layer can be installed in front of the piezoelectric element to protect the piezoelectric element and to match the impedance between the tested material and piezoelectric element. An encapsulation noise control material was installed between the casing and piezoelectric element to minimize the external noise, and furthermore, the electrical noise was reduced by grounding the casing. The disk-shaped piezoelectric transducers had an outer diameter of 25 mm, and the active element diameter was 19 mm. Considering that the Huygen’s wavelets radiated from all points on the surface of transducers propagate through the material, the amplitude fluctuates in the near field of the transducer [26]. The nominal frequency of the transducer used in this study was 50 kHz.

An impulse signal was generated by a pulser (JSR Ultrasonics, DPR300) to excite the source transducer, as shown in Figure 7b. The pulser was used to control the amplitude of the excitation pulse, which could be adjusted between 100 and 475 V. An elastic wave generated by the source traveled through the rock specimen and was then detected by the receiver transducer. The received signals were filtered by passing only frequencies ranging from 0.5 to 2.000 kHz and were amplified by using a filter-amplifier (Krohn-Hite, 3944). To remove the uncorrelated noise, the received signals were recorded by the oscilloscope, stacking 1024 signals. A pair of disk-shaped piezoelectric transducers was installed at the side of a rock specimen. For a good coupling, a vacuum grease was applied between the piezoelectric transducer and the rock specimen. The installed height and lateral position of the transducers correspond to the middle depth of the drilled hole and half of the hole-to-face distance, respectively. Note that the hole-to-face distance is defined as the minimum distance between the center of the hole bored in the rock specimen and one of the four side surfaces of the rock specimen. The received signals were measured every 10 s from the beginning of heating.

## 3. Results and Discussion

### 3.1. Elastic Wave Monitoring

**Figure 8:**
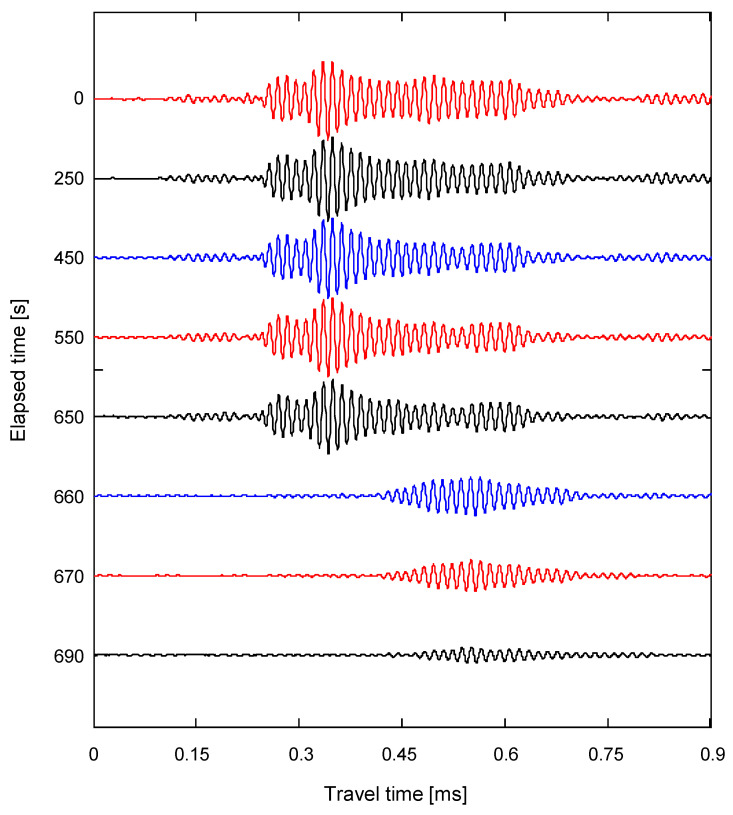
Typical signals obtained in a rock specimen from the wave transducers with the time elapsed.

The elapsed time after heating the system was recorded from 0 to 30 min, and the output signals were obtained from the elastic wave measurement system every 10 s. The typical waveforms obtained for a rock specimen with the elapsed time are plotted in Figure 8. Considering the radius of the transducer and the wavelength, the length of the rock specimen was significantly greater than the length of the near field. In the initial stage, the waveforms rarely changed with the elapsed time, and accordingly, the first arrival time and maximum amplitude of the waveforms obtained at initial times remained almost constant. As the crack propagated, the first arrival time increased rapidly, and the maximum amplitude gradually decreased as more time elapsed.

**Figure 9:**
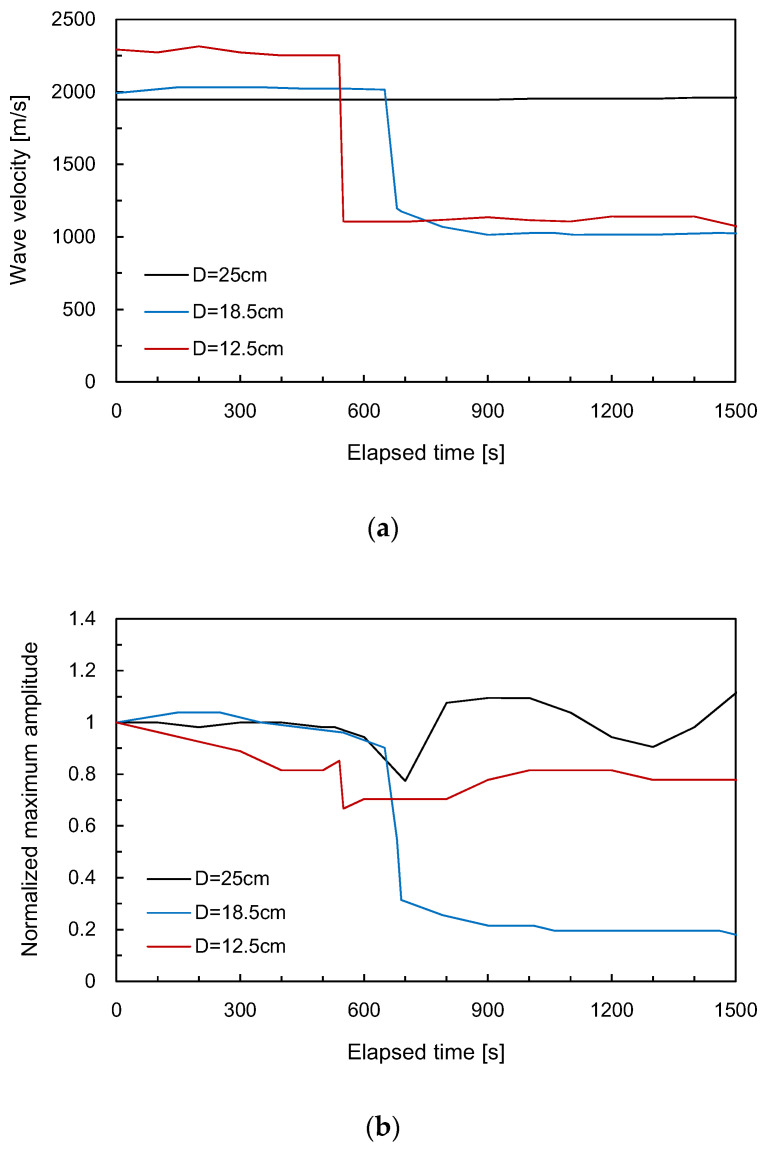
Variation in wave velocities and the maximum amplitudes along the elapsed time for the rock specimen: (**a**) wave velocity; (**b**) maximum amplitude.

The evolution of the shear wave velocity evaluated from the travel distance of the elastic wave and the first arrival time along the elapsed time is plotted in Figure 9a. Before heating, the values of the shear wave velocities for the hole-to-face distances of 12.5, 18.5, and 25 cm were 2294, 1992, and 1945 m/s, respectively, which were slightly less than the values obtained from the rock core samples. Considering the size of the specimen, the number of cracks in the rock specimen may be greater than that in the core samples, thereby reducing the elastic wave velocity. For the initial stage, the shear wave velocity remained almost constant, regardless of the hole-to-face distance. The values of the shear wave velocities for the hole-to-face distances of 12.5 and 18.5 cm decreased drastically for elapsed time of 540 and 650 s, respectively. As the crack propagated, the shear wave velocities for the hole-to-face distances of 12.5 and 18.5 cm reached around 1100 and 1000 m/s, respectively, because the crack damage causes a decrease in the elastic wave velocities [22]. For a hole-to-face distance of 25 cm, the crack was generated opposite to the transducers, as shown in Figure 6a, and constant shear wave velocity was maintained during the test. Note that for a hole-to-face distance of 25 cm, the heater was located at the center of the rock specimen. The direction-dependent expansion of the heater can lead to the crack propagating toward one or both of the two free faces in opposite directions. The high temperature induced by the heater rarely affected the variation in the shear wave velocity in the rock specimen with a hole-to-face distance of 25 cm. Nasseri et al. [27] reported that the compressional wave velocity estimated when heating a rock sample at room temperature to 850 °C gradually decreased. That means that the rock crack in the cuboid specimen was influenced by the mechanical expansion of the vermiculite with minimum heat transfer directly through the specimen.

The maximum amplitude measured for each output signal was normalized with that measured for the output signal before heating. Figure 9b shows the variation in the normalized maximum amplitudes with elapsed time. In the initial stage, the normalized maximum amplitudes for the hole-to-face distances of 12.5 and 18.5 cm gradually decreased and then rapidly dropped around the elapsed time of 540 and 650 s, respectively. After the drop with the maximum amplitude, the normalized maximum amplitudes remained almost constant with time. Interestingly, the normalized maximum amplitudes for the hole-to-face distance of 25 cm significantly dropped around 700 s after the stable amplitude variation at the initial stage. This decrease was probably because of crack propagation from the side opposite to the location of installation of the transducers.

### 3.2. Elapsed Time to Rock Fracture

**Figure 10:**
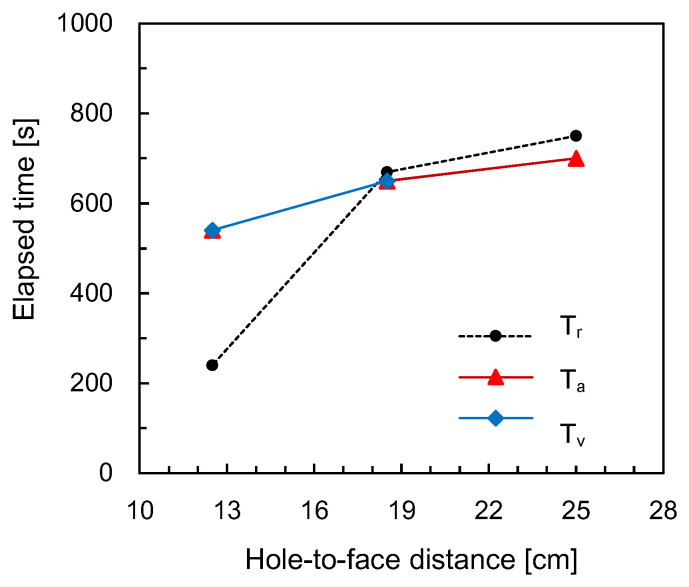
Variation in the elapsed time until crack propagation according to the hole-to-face distance. T_r_ denotes the elapsed time recorded by observation. T_v_ and T_a_ indicate the elapsed times until the decrease in the velocity and amplitude of elastic waves, respectively.

The surface of the rock specimen was observed by the naked eye during the expansion test to investigate crack propagation. For the three rock specimens, the crack propagation on the surface was located at the midpoint of the width of each specimen, as shown in Figure 6. The elapsed time at the moment that the crack was propagated on the surface of the rock specimen was recorded. The elapsed time corresponding to crack propagation (*T_r_*) along the hole-to-face distance is plotted in Figure 10. For the hole-to-face distance of 25 cm, the elapsed time corresponding to the crack propagation was 750 s. The elapsed time corresponding to crack propagation decreased with a decrease in the hole-to-face distance. For a hole-to-face distance of 12.5 cm, the crack generated on the surface of the rock specimen was detected at 240 s.

The elapsed times until the fall in the velocity and amplitude of the shear waves (*T_v_* and *T_a_*) are also plotted in Figure 10. The elapsed times until the decrease in the velocity and amplitude of shear waves were almost equal, except for the hole-to-face distance of 25 cm. Note that there was no decrease in the shear wave velocity for the hole-to-face distance of 25 cm because in this case, crack propagation was initiated on the specimen on the side opposite to the transducers. In general, the elapsed times until both the velocity and amplitude of shear waves fell decreased with a reduction in the hole-to-face distance. For the hole-to-face distances of 18.5 and 25 cm, the elapsed times until both velocity and amplitude of shear waves reduced were almost equal to those until crack propagation was detected on the surface of the rock specimens. For a hole-to-face distance of 12.5 cm, the elapsed times until both velocity and amplitude of shear waves reduced were significantly greater than those until crack propagation was detected on the surface of the specimen. Considering that the transducers were located at the middle depth of the drilled hole, the crack for a hole-to-face distance of 12.5 cm was propagated clearly from the surface to the bulk of the rock specimen, while the crack propagation for the hole-to-face distances of 18.5 and 25 cm was detected almost simultaneously. Thus, the proposed shear wave monitoring system with the piezoelectric transducers may be useful for detecting crack propagation when the heater system was applied to the rock mass.

## 4. Conclusions

A new vibration-free excavation method based on vermiculite expansion for rock fracture was proposed in this study. The vermiculite-based filling material and rectangular heater rod were developed to improve the transfer of mechanical expansion from the borehole to the rock specimen. A modified heating system using a transformer resulted in both lower voltage for safety and higher current for rapid heating of the heater rod. The rectangular heater rod is durable at high temperatures owing to its large surface area and can be used to control the direction of rock cracking due to the difference in expansion pressure depending on the direction. Expansion tests were performed using the heating system and filling material for three large rock specimens with different hole-to-face distances. During the expansion tests, crack propagation was monitored by an elastic wave measurement system. For the two small hole-to-face distances, the wave velocity and maximum amplitude at the initial stage remained almost constant and then decreased after a certain elapsed time. For the greatest hole-to-face distance, shear wave velocity did not decrease, whereas the maximum amplitudes decreased after a certain time. The constant shear wave velocity maintained during the expansion test demonstrated that the rock crack in the specimen was influenced mainly by the mechanical expansion of vermiculite with minimal heat transfer from the heater at a high temperature. The elapsed times until the decrease in velocity and amplitude of shear waves reasonably corresponded to those until the crack propagation was detected on the surface of the rock specimens. In conclusion, the new excavation method with the expansion-induced materials has many advantages over other vibration-free methods in terms of rapid expansion, affordability, and crack control. The elastic wave monitoring system with the piezoelectric transducers may be effectively used to detect crack propagation when applying the heater system to a rock mass.

## References

[1] Park D., Jeon B., Jeon S. (2009). A numerical study on the screening of blast-induced waves for reducing ground vibration. Rock Mech. Rock Eng..

[2] Jeng F.S., Huang T.H., Hilmersson S. (2004). New development of waterjet technology for tunnel excavation purposes. Tunnelling and Underground Space Technology. Underground Space for Sustainable Urban Development, Proceedings of the 30th ITA-AITES World Tunnel Congress, Singapore, 22–27 May 2004.

[3] Song K.I., Oh T.M., Cho G.C. (2014). Precutting of tunnel perimeter for reducing blasting-induced vibration and damaged zone—Numerical analysis. KSCE J. Civ. Eng..

[4] Kim J.G., Song J.J. (2015). Abrasive water jet cutting methods for reducing blast-induced ground vibration in tunnel excavation. Int. J. Rock Mech. Min. Sci..

[5] Gustafsson N. (2011). Wire Cutting as a Complement to Drill and Blast in Vibration Sensitive Environments. Master’s Thesis.

[6] Lee J.S., Ahn S.K., Sagong M. (2016). Attenuation of blast vibration in tunneling using a pre-cut discontinuity. Tunn. Undergr. Space Technol..

[7] Lee J.H., Ahn S.K., Lee K.C., Bang C.S., Cho J.H., Sagong M. (2017). Wire saw cutting model development and performance investigation for vibration reduced tunnel excavation. Tunn. Undergr. Space Technol..

[8] Hiller D. The prediction and mitigation of vibration impacts of tunneling. Proceedings of the Acoustics—Breaking New Ground 2011.

[9] Jana S. (1991). Non-explosive expanding agent–an aid for reducing environmental pollution in mines. Indian Min. Eng. J..

[10] Arshadnejad S., Goshtasbi K., Aghazadeh J. (2011). A model to determine hole spacing in the rock fracture process by non-explosive expansion material. Int. J. Miner. Metall. Mater..

[11] Arshadnejad S., Goshtasbi K., Aghazadeh J. (2009). Stress concentration analysis between two neighboring circular holes under internal pressure of a non-explosive expansion material. Earth Sci..

[12] Ahn C.H., Hu J.W. (2015). Investigation of key parameters of rock cracking using the expansion of vermiculite materia. Materials.

[13] Walker G.F., Brindley G.W. (1951). Vermiculites and Some Related Mixed-Layer Minerals. X-ray Identification and Crystal Structures of Clay Minerals.

[14] Hillier S., Marwa E.M.M., Rice C.M. (2013). On the mechanism of exfoliation of ‘Vermiculite’. Clay Miner..

[15] Ahn C.H., Hu J.W. (2016). Experimental field tests and finite element analyses for rock cracking using the expansion of vermiculite materials. Adv. Mater. Sci. Eng..

[16] Schubnel A., Nishizawa O., Masuda K., Lei X.J., Xue Z., Guéguen Y. (2003). Velocity Measurements and Crack Density Determination during Wet Triaxial Experiments on Oshima and Toki Granites. Thermo-Hydro-Mechanical Coupling in Fractured Rock.

[17] Byun J.H., Lee J.S., Park K., Yoon H.K. (2015). Prediction of crack density in porous-cracked rocks from elastic wave velocities. J. Appl. Geophys..

[18] Byun Y.H., Han W., Tutumluer E., Lee J.S. (2016). Elastic wave characterization of controlled low-strength material using embedded piezoelectric transducers. Constr. Build. Mater..

[19] Byun Y.H., Tutumluer E. (2017). Bender elements successfully quantified stiffness enhancement provided by geogrid–aggregate interlock. Transp. Res. Rec..

[20] Byun Y.H., Tutumluer E., Feng B., Kim J.H., Wayne M.H. (2019). Horizontal stiffness evaluation of geogrid-stabilized aggregate using shear wave transducers. Geotext. Geomembr..

[21] Lee I.M., Truong Q.H., Kim D.H., Lee J.S. (2009). Discontinuity detection ahead of a tunnel face utilizing ultrasonic reflection: Laboratory scale application. Tunn. Undergr. Space Technol..

[22] Schubnel A., Benson P.M., Thompson B.D., Hazzard J.F., Young R.P. (2006). Quantifying Damage, Saturation and Anisotropy in Cracked Rocks by Inverting Elastic Wave Velocities. Rock Damage and Fluid Transport: Part I..

[23] Walsh J.B. (1965). The effect of cracks on the compressibility of rock. J. Geophys. Res..

[24] O’Connell R.J., Budiansky B. (1974). Seismic velocities in dry and saturated cracked solids. J. Geophys. Res..

[25] Feng J., Liu M., Fu L., Zhang K., Xie Z., Shi D., Ma X. (2020). Enhancement and mechanism of vermiculite thermal expansion modified by sodium ions. RSC Adv..

[26] Lee J.S., Santamarina J.C. (2005). P-wave reflection imaging. Geotech. Test. J..

[27] Nasseri M.H.B., Schubnel A., Young R.P. (2007). Coupled evolutions of fracture toughness and elastic wave velocities at high crack density in thermally treated Westerly granite. Int. J. Rock Mech. Min. Sci..

